# Aberrant super-enhancer-driven oncogene ENC1 promotes the radio-resistance of breast carcinoma

**DOI:** 10.1038/s41419-021-04060-5

**Published:** 2021-08-06

**Authors:** Lin Li, Nan Wang, Mingzhi Zhu, Youyi Xiong, Fang Wang, Guangcheng Guo, Xinxing Wang, Yuanyan Gu

**Affiliations:** Department of Breast Surgery, the First Affiliated Hospital of Zhengzhou University, Zhengzhou, 450052 Henan People’s Republic of China

**Keywords:** Breast cancer, Breast cancer

## Abstract

Poor response of tumors to radiotherapy is a major clinical obstacle. Because of the dynamic characteristics of the epigenome, identification of possible epigenetic modifiers may be beneficial to confer radio-sensitivity. This research was set to examine the modulation of ectodermal-neural cortex 1 (ENC1) in radio-resistance in breast carcinoma (BC). In silico identification and immunohistochemical staining revealed that overexpression of ENC1 promoted BC metastasis to the bone and brain. Moreover, its overexpression promoted the translocation of YAP1/TAZ into the nucleus and enhanced expression of GLI1, CTGF, and FGF1 through the Hippo pathway. ENC1 expression was controlled by a ~10-kb long SE. ENC1-SE^distal^ deletion reduced ENC1 expression and inhibited the malignant behavior of BC cells and their resistance to radiotherapy. The binding sites on the ENC1-SE region enriched the shared sequence between TCF4 and ENC1 promoter. Knocking-down TCF4 inhibited luciferase activity and H3K27ac-enriched binding of the ENC1-SE region. Additionally, SE-driven ENC1 overexpression mediated by TCF4 may have clinical implications in radio-resistance in BC patients. Our findings indicated that ENC1 overexpression is mediated by SE and the downstream TCF4 to potentiate the Hippo/YAP1/TAZ pathway. Targeting this axis might be a therapeutic strategy for overcoming BC radio-resistance.

## Introduction

Breast carcinoma (BC) remains a key health problem with an incidence of approximately 17,000,000 new diagnoses each year, suggesting slow progress made in the prevention [1]. The treatment opportunities for metastatic BC have improved over the years as our knowledge of the interplays of signaling pathways and comprehension of biological behaviors has advanced [2]. Still, the current therapeutic options for BC mainly include combination of surgical reduction and local radiation with anticancer drugs; while a number of patients ultimately develop into more aggressive malignant forms that are resistant to the most frequent treatments [3]. Thus, identification of novel predictive and prognostic biomarkers in guiding the clinical treatment for BC is of great importance to the management of radio-resistance in BC.

Epigenetics is the study of heritable alterations in the phenotype that does not include any alteration in DNA sequence, and the words “epigenesis” and “genetics” were coined by Conrad H. Waddington in 1942 to describe the “causal mechanisms” through which “the genes of the genotype bring about phenotypic effects” [4]. Classical epigenetic mechanisms involve histone modification, chromatin remodeling, as well as DNA methylation [5]. In the present study, our meta-analysis revealed that ectodermal-neural cortex 1 (ENC1) is significantly upregulated in BC and its overexpression is linked to responses to radio-therapy. In line with our prediction, ENC1 has been lately emphasized as a possible prognostic and metastasis-related biomarker of BC, and may serve as an attractive therapeutic target against BC [6]. Still, the underlying mechanism of action remains largely unknown. Super-enhancers (SE) are clusters of transcription enhancers that elevate expression profiles of genes, and tumor cells produce SE at oncogenes and other genes, which is important in tumor pathogenesis [7]. SE differ from typical enhancers regarding size, transcription factor content, ability to induce transcription, and sensitivity to perturbation [8]. Oncogenic SE were found in a wide spectrum of malignancies, including neuroblastoma, lung cancer, esophageal cancer, gastric cancers, as well as BC [9]. For instance, HCCL5 was transcriptionally driven by a transcription factor ZEB1 via SEs and was significantly overexpressed in human hepatocellular carcinoma tissues, correlating with dismal overall survival of patients [10]. More relevantly, ANLN was identified by epigenomic and transcriptomic profiling as a triple-negative BC-specific gene regulated by SE [11]. Therefore, we assumed that the oncogenic role of ENC1 played in BC is elicited through the SE and an associated transcription factor. Our discoveries might offer a new potential target for BC prognosis and treatment.

## Materials and methods

### In silico identification

Finak breast [12], Sorlie breast [13], and Sorlie breast 2 [14] were studied by setting the primary filter condition to cancer vs normal analysis radiotherapy response status-recurrence at 3 years. We obtained a series of genes most associated with recurrence within 3 years in BC patients treated with radiotherapy. The ENC1 expression and the local recurrence-free survival (LRFS) rate of BC patients who have received radiotherapy were obtained from the breast cancer datasets Servant [15], Vande Vijver [16], and Wang [17], followed by correlation analyses. Subsequently, the Ivshina breast cancer dataset [18], the Schmidt breast dataset [19], and the Esserman breast dataset [20] were accessed from the Oncomine database and subjected to correlation analyses with the ENC1 expression. Genes with Spearman correlation coefficients greater than 0.45 were collected for enrichment analysis using a reactome signaling pathway analysis. Bubble plots were plotted by R ggplot2 package. The correlation between the level of immune cell infiltration in tumor tissues and the ENC1 or TCF4 expression in the TCGA-BRCA database was analyzed using the R cibersort package.

### Sample collection

Between September 2006 and October 2017, 91 samples of female patients with BC were collected from the First Affiliated Hospital of Zhengzhou University. Of these 91 patients, all underwent postoperative radiotherapy, and local recurrence and metastasis occurred in 39 cases. Two pathologists independently verified the pathological diagnoses. Non-responders were defined as those who had local recurrence in the breast and/or lymph nodes after completion of radiotherapy. The study was approved by the Ethical Committee of the First Affiliated Hospital of Zhengzhou University and all procedures were conducted according to the *Declaration of Helsinki*. All of the patients provided written informed consent.

### RT-qPCR analysis

Total RNA was isolated from tissues or cultured cells using Trizol, and 1 µg RNA was reversely transcribed into cDNA using a Superscript First-Strand cDNA Synthesis Kit (18080-051, Invitrogen Inc., Carlsbad, CA, USA). RT-qPCR analysis was performed on the LightCycler 480 System (Roche Diagnostics, Co., Ltd., Rotkreuz, Switzerland) using the SYBR Premix Ex Taq II kit (DRR081A, Takara Biotechnology Ltd., Dalian, Liaoning, China). PCR primers used are indicated in Supplementary Table S1. The relative expression was calculated using 2^−ΔΔCt^ [21].

### Western blot

Protein levels were determined using a BCA protein assay kit (Thermo Fisher Scientific Inc., Waltham, MA, USA) as per the manufacturer’s protocol, and equal amounts of protein were separated by sodium dodecyl sulfate (SDS)-polyacrylamide gel electrophoresis as previously described [22]. After transferring proteins from the gel to the polyvinylidene fluoride membranes, the blot was blocked with 5% skim milk in phosphate-buffered saline/Tween (PBST) for 1 h at ambient temperature and then incubated with different antibodies (Supplementary Table S2). Protein expression was detected using an HRP-conjugated secondary rabbit antibody to IgG and an enhanced chemiluminescence detection system.

### Histological staining

The expression of ENC1 or TCF4 in paraffin-embedded tumor samples was checked using an immunohistochemistry kit (Roche) according to the manufacturer’s instructions. Briefly, tumor slides were treated with pepsin for 10 min and incubated with digoxin (DIG)-labeled monoclonal antibody (RiboBio, Guangzhou, Guangdong, China) for 4 h (both at ambient temperature). After three washes (5 min) with PBST and sealing with 10% FBS for 30 min, the sections were incubated overnight at 4 °C with anti-DIG antibody. Subsequently, the sections were treated with HRP-conjugated anti-rabbit IgG for 1 h. After the addition of diaminobenzidine and hematoxylin, the sections were finally observed under an optical microscope. The expression of ENC1 or TCF4 was analyzed by combining the percentage of positively stained tumor cells with the intensity of positive staining. Staining intensity was graded as follows: 0, no staining; 1, weak staining (light purple); 2, moderate staining (purple-dark purple); 3, strong staining (dark purple) as described by Cerilli et al. [23].

### Clustered regularly interspaced short palindromic repeat (CRISPR)/Cas9-mediated SE repression

The CRISPR-Cas9 platform was utilized as explained previously [24]. To design CRISPR constructs for SE depletion, the sequence 5′-ACCTACCCTTTGGCCTACGTAC-3′ targeting the left SE of human ENC1, and the sequence 5′-GGGCTGAGTAGAATGGGCG-3′ targeting the right SE of human ENC1, were cloned into the LentiCRISPR (pXPR_001) plasmid. LentiCRISPR plasmid served as a NC. To corroborate the detection efficiency, trans-SE PCR primers that were located on the outer side of the CRISPR single guide RNA (sgRNA) were designed and amplified with a region of approximately 20 kb^+^. Assumed efficient CRISPR cutting and DNA repair via non-homologous end joining, it is expected that approximately 320 bp of product will be generated. The following ENC1^distal^ PCR deletions across primers were used: F: 5′-AGGGGATCACCTGTCTGT-3′; R: 5′-TCCTGACCACAGG TGATCCG-3′.

### Cell culture and treatment

MDA-MB-231 and BT549 cells were from the American Type Culture Collection (ATCC, Manassas, VA, USA), while normal human mammary epithelial cells HB-2 and MCF10A were from China Center for Type Culture Collection (Wuhan, Hubei, China). All the cell lines were grown in DMEM containing 10% FBS at 37 °C in 5% CO_2_. Radio-resistant cells (MDA-MB-231/RaR and BT549/RaR) were established by exposing MDA-MB-231 and BT549 cells to 2 Gy gamma irradiation twice a week. Cell survival was analyzed using the linear quadratic model and single-hit multitarget model, and cells with a death rate less than 10% under radiation were identified as radio-resistant cells.

### Stable silencing and overexpression of ENC1

MDA-MB-231 and BT549 parental (PA) or RaR cells were seeded in a 6-well plate at 50,000 cells per well and incubated in 2 mL DMEM containing 10% FBS overnight. Subsequently, the medium was refreshed with fresh medium. Control (ctrl) small interfering RNA (si), siRNA targeting ENC1 #1 (siENC1-#1) or #2 (siENC1-#2) complexes were added to silence ENC1 expression in RaR cells. After 24 h, the cells were rinsed with PBS solution and incubated in fresh medium for an additional 48 h. For ENC1 overexpression experiments, parental MDA-MB-231, BT549, and MCF10A (#CRL-10317, ATCC) and HB-2 (#151441, Ximbio, London, UK) cells were plated in 6-well plates at 50,000 cells per well and incubated with Lipo2k/plasmid complexes overnight. After PBS washes, the cells were incubated in fresh medium for an additional 48 h. After transfection, the cells were screened in 5 μg/mL G418 medium. After 3 days of culture, cells were maintained at 1.5 μg/mL G418. After verification of success ENC1 expression alteration using RT-qPCR, the cells were subjected to flow cytometry analysis, migration and invasion analysis, and western blot analysis.

### Colony formation assay

MDA-MB-231/RaR, BT549/RaR, MDA-MB-231/PA and BT549/PA cells were seeded in 6-well plates at 50,000 cells per well and incubated in 2 mL DMEM containing 10% FBS for 24 h. After medium replacement, ENC1 expression was silenced in MDA-MB-231/RaR and BT549/RaR or overexpressed in MDA-MB-231/PA and BT549/PA cells. Subsequently, cells were plated in 6-well plates and irradiated with various doses of gamma radiation for 2 h. The number of colonies formed was counted using crystal violet staining.

### Apoptosis detection

MDA-MB-231/RaR, BT549/RaR, MDA-MB-231/PA and BT549/PA cells were treated as aforementioned. Subsequently, the cells were irradiated with 2 Gy gamma radiation, detached with trypsin and stained with Annexin V-fluorescein isothiocyanate (FITC, 20 µg/mL) and propidium iodide (PI, 50 µg/mL) for flow cytometry analysis using a Cytomics FC 500 instrument (Beckman Coulter, Inc., Chaska, MN, USA) [25].

### Transwell assay

For migration assay, cells were plated into the apical chamber of 24-well Boyden plate (8 µM; Corning Glass Works, Corning, N.Y., USA). For invasion assay, cells were added into the apical chambers precoated with Matrigel [26]. MCF10A and HB-2 cells overexpressing ENC1 were first cultured. Subsequently, the cells were detached with trypsin and transferred to the apical chambers (1000 cells per well) filling with FBS-free DMEM. After 24 h, cells attached to the bottom of the apical chamber were counted and imaged to show cell migration and invasion.

### Animal experiments

All animal procedures were permitted by Animal Care and Use Committee of the First Affiliated Hospital of Zhengzhou University. Adequate measures were taken to minimize the number of animals used and to ensure minimal pain or discomfort. Two hundred-twenty female BALB/c nude mice (6 to 8 weeks old) were obtained from Hunan SJA Laboratory Animal Co., Ltd. (Changsha, Hunan, China). Luciferase-labeled PA cells were injected into the left ventricle of anesthetized female nude mice. After intraperitoneal injection of D-fluorescein (PerkinElmer) at 75 mg/kg, the in vivo metastases were monitored by measuring the photon flux of bioluminescence imaging (BLI) signals in the mice. BLI was acquired with an IVIS imaging system (Xenogen Corporation, Alameda, CA, USA) 2–5 min after injection. BLI signal data were obtained after background deduction and standardized to signals obtained immediately after xenografting (day 0) [27].

For in vivo tumor growth experiments, tumor xenografts were established by subcutaneously injecting 2 × 10^6^ tumor cells with Matrigel (1:1) into the nude mice [28]. Tumor volume and weight of the mice were observed. Tumor size was measured using a slide caliper (volume = 0.5 × a × b^2^, where a is the length and b is the width). After euthanasia of the nude mice, the tumor tissues were removed and weighed.

### Terminal deoxynucleotidyl transferase (TdT)-mediated 2’-Deoxyuridine 5’-Triphosphate (dUTP) nick end labeling (TUNEL)

Cryopreserved tissue sections were fixed with 4% polyformaldehydes, permeabilized with 0.3% Triton X-100 in PBS, and blocked with endogenous peroxidase blocking buffer. The sections were treated with the TUNEL reaction mixture (Roche) for 60 min and incubated with Converter-peroxidase (Roche) at 37 °C for 0.5 h. The sections were colored with diaminobenzidine substrate (Servicebio, Wuhan, Hubei, China) and counter-stained with hematoxylin. The proportion of apoptotic cells was determined under an optical microscopy by counting TUNEL-positive cells and total cells in four fields of view (×400) [29].

### Chromatin immunoprecipitation (ChIP)

A ChIP analysis kit (#53008, Active Motif., Carlsbad, CA, USA) was applied as per the manufacturer’s protocol [30]. The cells were cross-linked with 1% formaldehyde for 10 min at ambient temperature and neutralized with glycine for 5 min. The cells were resuspended in SDS buffer and subjected to sonication. After centrifugation, the harvested supernatant was diluted in IP dilution buffer. Antibodies against TCF4 (Cat#13440 S, Cell Signaling Technologies, Beverly, MA, USA), acetylation at H3 lysine 27 (H3K27ac, GTX128944, GeneTex, Inc., Alton Pkwy Irvine, CA, USA) or IgG (Cat#2729S, Cell Signaling Technologies) were utilized. After IP, protein A-agarose was supplemented for another 1-h incubation. The precipitates were washed and used for DNA purification by RT-qPCR after de-crosslinking. The primers used are listed below: ENC1-Promoter ChIP1: 5′-GAATCGATCCTGATAGCTA-3′, ENC1-promoter ChIP P2: 5′-GATTCGTGTAACCCTGTCT-3′; ENC1-SE ChIP P1: 5′-CCACCTACGTGTCGATCGATCCGA-3′; ENC1-SE ChIP P2: 5′-CTGTGCAAATCGATCCGTAGTTGCA-3′.

### Luciferase report assay

For the ENC1-P luciferase reporter gene, the 1851-bp region of the ENC1 promoter was subcloned into the XhoI and HindIII sites of the pGL3-Basica vector (Promega Corporation, Madison, WI, USA). To generate the ENC1 promoter enhancer (PE) luciferase reporter genes, the 1899-bp region of the ENC1-E1 enhancer, the 1838-bp region of the ENC1-E2 enhancer, the 1533-bp region of the ENC1-E3 enhancer, and the 1999-bp region of the ENC1-E4 enhancer were subcloned into ENC1. The luciferase reporter genes ENC1-E1, ENC1-E2, ENC1-E3, and ENC1-E4 plasmids were generated using 4-Promoter luciferase reporter gene with the SalI/BamHI restriction enzyme site. To confirm the TCF4 binding sites on the ENC1-E2 enhancer, a 241 bp region containing the wild-type (WT) TCF4 motif-binding sequence (from chr5: 74687892-74684243) was inserted into the SalI and BamHI sites of the pGL4 vector (downstream of the luciferase) and the minimal promoter (Promega) to produce the TCF4 luciferase reporter gene (pGL4-WT). To generate the pGL4-mutant (Mut) plasmid, mutant TCF4 binding sites from GTGGTGGTTT to GTGAACGTT were constructed by PCR-based site-directed mutagenesis.

### Statistical analysis

All statistical data were displayed as means ± standard deviation (SD) and were analyzed using SPSS 20.0 (IBM Corp. Armonk, N.Y., USA). Survival was analyzed using Kaplan–Meier method and evaluated using the log-rank test and univariate Cox proportional risk regression to identify hazard ratio (HR) and 95% confidence interval (CI). A Cox proportional risk regression model was utilized to identify independent prognostic factors with the help of multivariate analysis. The connection between TCF4 expression and ENC1 expression was evaluated using Spearman’s correlation analysis. The significance of the differences among three or more groups was conducted by using one-way or two-way ANOVA with Tukey post-test. All plots were drawn by using GraphPad Prism 8.0.1 software (GraphPad, San Diego, CA, USA). *p*-Values less than 0.05 were deemed as statistically significant.

## Results

### ENC1 is significantly overexpressed in BC tissues and is strongly associated with the radiation responses of patients

**Fig. 1:**
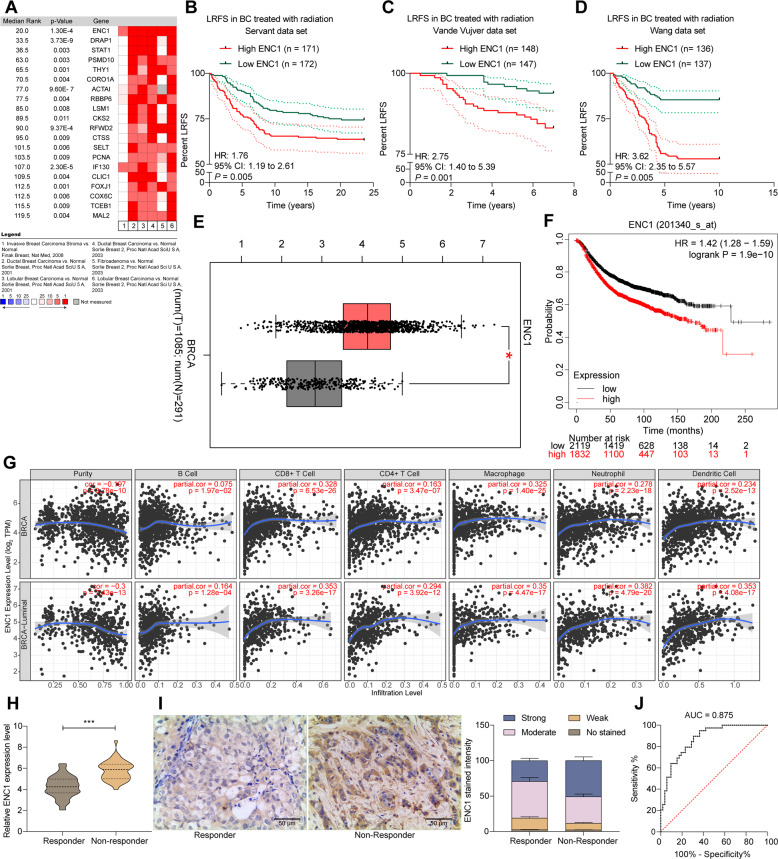
ENC1 is significantly overexpressed in BC tissues and is strongly associated with radiation responses of patients. **A** The oncomine database to detect the genes most associated with BC recurrence within three years after radio-resistance in the Finak Breast, Sorlie Breast, Sorlie Breast 2 datasets. **B**–**D** Correlation between ENC1 expression and LRFS in BC patients in Servant (**B**), Vande Vijver (**C**), and Wang (**D**) datasets. **E** ENC1 expression in the TCGA-BRCA dataset. **F** Analysis of the association between ENC1 expression and survival of patients in TCGA-BRCA dataset. **G** Analysis of the connection between ENC1 expression and the infiltration of immune cells in BC patients. **H**, **I** RT-qPCR (**H**) and immunohistochemical detection (**I**) of ENC1 expression in 39 non-responder and 52 responder BC patients. **J** ROC curve analysis of the predictive efficacy of ENC1 expression and sensitivity to radiotherapy in BC patients. All the data are expressed as the mean ± SD. Unpaired *t*-test (panel **H**) or two-way ANOVA with Tukey’s multiple comparison test (panel **I**) were utilized to detect significant differences between data, ***p < 0.001.

By setting the Primary Filter condition to cancer vs normal analysis radiotherapy response status-recurrence at 3 years, we obtained a series of genes most relevant to recurrence within 3 years in BC patients treated with radiotherapy. The heatmap in Fig. 1A shows some of the differentially expressed genes. ENC1 was revealed to be significantly upregulated in all six datasets. We observed that ENC1 had the highest expression in Finak Breast [12], Sorlie Breast [13], and Sorlie Breast 2 [14]. We further analyzed the LRFS of BC patients after radiotherapy in the BC datasets Servant [15], Vande Vijver [16], and Wang [17]. The patients with poor expression of ENC1 had a higher survival rate after radiotherapy in three datasets (Fig. 1B–D). Our subsequent analysis in TCGA-BRCA database revealed a significant augment in ENC1 expression in cancer tissues (Fig. 1E). Consistent with the results of Fig. 1B–D, the patients with elevated ENC1 in the TCGA database had a lower survival rate (Fig. 1F). We found significant correlations between ENC1 overexpression and the infiltration of CD8^+^ T cells, macrophages, neutrophils, and dendritic cells in BC tissues (Fig. 1G).

To further clarify the correlation between ENC1 and radio-resistance in BC patients, we collected 91 BC samples from patients, 39 of whom were radiotherapy non-responder and the remaining 52 of whom were radiotherapy responder. The expression or staining intensity of ENC1 was much higher in non-responders than that in the responders, as RT-qPCR and immunohistochemistry revealed (Fig. 1H, I). Moreover, relative operating characteristic (ROC) curves determined the predictive efficacy of ENC1 expression for radiotherapy in BC patients (Fig. 1J).

### ENC1 confers oncogenesis and radio-resistance to BC cells

Further, the expression of ENC1 in the TCGA database was remarkably elevated in multiple cancers (Supplementary Fig. S1A). The staining intensity of ENC1 in BC tissues was also enhanced versus that in normal breast tissues in the Human Protein atlas database (Supplementary Fig. S1B). To explore the association between ENC1 expression and breast carcinogenesis, we transfected ENC1 overexpression plasmids into normal breast epithelial cells HB-2 and MCF10A, and confirmed successful transfection (Supplementary Fig. S1C) by western blot. We found that the cell morphology of HB-2 and MCF10A changed more markedly from epithelial to mesenchymal morphology after overexpression of ENC1 (Supplementary Fig. S1D). Moreover, a significant increase was observed in cell activity after overexpression of ENC1 (Supplementary Fig. S1E). The proportion of apoptotic cells showed a declining trend (Supplementary Fig. S1F). The invasion and migration capacities of MCF10 and HB-2 cells in vitro were also significantly increased after overexpression of ENC1 (Supplementary Fig. S1G, H). The above results indicate that ENC1 has a significant role in the development of BC.

**Fig. 2:**
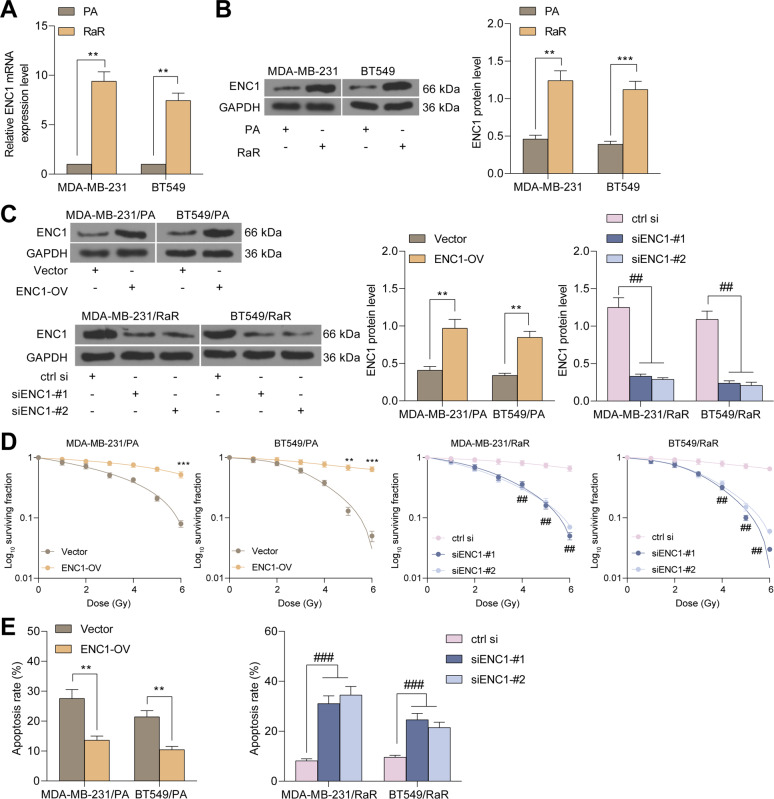
ENC1 confers oncogenesis and radio-resistance to BC cells. **A**, **B** RT-qPCR (**A**) and western blot (**B**) detection of mRNA and protein expression of ENC1 in PA and RaR cells. ENC1 was overexpressed in PA cells and knocked-down in RaR cells. **C** Western blot detection of transfection efficiency. **D** Cell viability of MDA-MB-231 and BT549 cells after 2 h of exposure to different doses of gamma radiations measured by colony formation assay. **E** Proportion of apoptotic cells detected by flow cytometry after irradiation of PA and RaR cells with 2 Gy doses of radiation. Data are representative of 3 separate experiments performed in triplicate. All the data are expressed as the mean ± SD. Two-way ANOVA with Tukey’s multiple comparison test were utilized to detect significant differences between data. **p < 0.01, ***p < 0.001; ^##^*p* < 0.01; ^###^*p* < 0.001.

To investigate the impact of ENC1 on the sensitivity of BC cells to radiotherapy, we constructed radio-resistant BC cell lines MDA-MB-231/RaR and BT549/RaR. We first assessed the ENC1 expression in the cells and noted that its expression was significantly higher in RaR cells than that in PA cells (Fig. 2A, B). Thereafter, we overexpressed ENC1 in PA cells, while knocked down ENC1 expression in RaR cells (Fig. 2C). Subsequently, we found that RaR cells had significantly increased sensitivity to radiotherapy, while PA cells had significantly increased resistance to radiotherapy after transfection (Fig. 2D). Furthermore, we used a 2 Gy dose of gamma radiation to irradiate PA and RaR cells, and we found that after overexpression of ENC1, the rate of apoptosis was significantly reduced in PA cells and increased in RaR cells after irradiation (Fig. 2E).

**Fig. 3:**
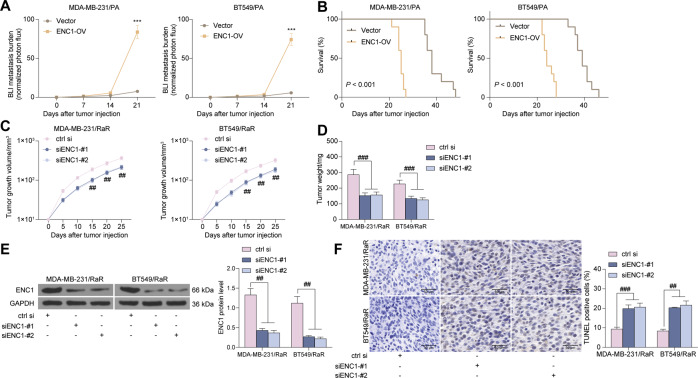
ENC1 knockdown sensitizes BC cells to radiotherapy in vivo. **A** The metastatic ability of MDA-MB-231/PA and BT549/PA cells overexpressing ENC1 in mice (*n* = 6). **B** analysis of the survival of mice after injection of MDA-MB-231 and BT549 cells (*n* = 10). **C**, **D** tumor growth curve (**C**) and weight (**D**) of nude mice injected with MDA-MB-231 and BT549 cells with ENC1 knockdown (*n* = 6). **E** ENC1 protein expression in tumor tissues examined using western blot. **F** Apoptosis rate in xenograft tumors examined by TUNEL staining. All the data are expressed as the mean ± SD. Two-way ANOVA with Tukey’s multiple comparison test were utilized to detect significant differences between data. ***p < 0.001; ^##^*p* < 0.01, ^###^*p* < 0.001.

Next, we established a metastatic animal model by intracardiac injection of parental MDA-MB-231 and BT549 cells overexpressing ENC1 and stably expressing the firefly luciferase reporter, and radiated mice at 4 Gy of gamma every three days. Mice overexpressing ENC1 had significantly increased tumor burden compared to mice carrying vector cells, as detected by BLI signal detection in the brain and hindlimb (Fig. 3A). It was worth noting that the survival of mice carrying ENC1 overexpressing cells was significantly reduced (Fig. 3B).

We also found that knocking down ENC1 significantly promoted the sensitivity of RaR cells to radiotherapy in vivo and suppressed tumor growth rate (Fig. 3C, D). Since we used siRNAs to knock down the expression of ENC1 in cells, and the effectiveness of siRNAs decreases with cell replication, we used western blot to detect the expression of ENC1 in tumor tissues, and we found that siENC1 still has the ability to reduce the expression of ENC1 (Fig. 3E). Moreover, silencing of ENC1 also enhanced apoptosis rate of tumor tissues after radiotherapy (Fig. 3F). Together, these observations suggest that silencing of ENC1 reduces the malignant biological behavior of BC cells as well as promotes sensitivity to radiotherapy.

### ENC1 is a SE-driven gene

**Fig. 4:**
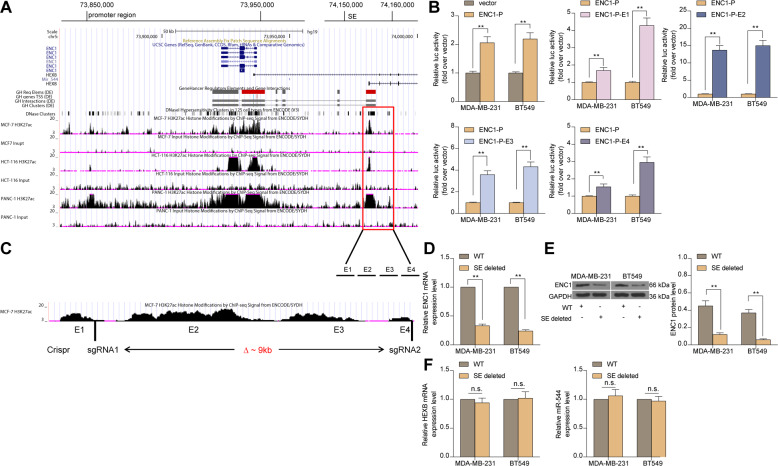
ENC1 is a SE-driven gene. **A** H3K27ac levels of ENC1 were examined using the UCSC genome browser. **B** The luciferase activity of ENC1-P, ENC1-E1, ENC1-E2, ENC1-E3 and ENC1-E4 in MDAMB-231 and BT549 cells measured by luciferase report assay. **C** The location of two sgRNAs flanking the 9 kb ENC1-SE^distal^. **D**, **E** RT-qPCR (**D**) and western blot detection (**E**) of ENC1 mRNA and protein expression in MDA-MB-231 and BT549 cells after SE deletion. **F** RT-qPCR for the expression of HEXB and miR-544, located in the same chromosomal region of ENC1, in MDA-MB-231 and BT549 cells after SE deletion. Data are representative of 3 separate experiments performed in triplicate. All the data are expressed as the mean ± SD. Two-way ANOVA (panel B, D-F) with Tukey’s multiple comparison test were utilized to detect significant differences between data. **p < 0.01, ns, nonsignificant.

To examine the transcriptional regulation of ENC1, we accessed publicly available ChIP-seq data for H3K27ac using the UCSC genome browser (http://bio.lundberg.gu.se/courses/vt13/ucsc.html) and the mRNA-seq data from the ENCODE website (https://www.encodeproject.org/). Interestingly, by sequencing the SE within the gene-desert region based on H3K27ac enrichment, we identified an approximately 10-kb SE region of ENC1 (ENC1-SE^distal^, a region approximately 60 kb upstream of ENC1) in MCF-7 (human BC cell line), HCT116 (human bladder cell line), and PANC-1 (human pancreatic cancer cell line). Subsequently, we divided the SE region into four parts (E1 to E4) (Fig. 4A). The luciferase reporter vectors for ENC1-P (containing the ENC1 promoter sequence) as well as ENC1-E1, ENC1-E2, ENC1-E3 and ENC1-E4 were constructed. We found that luciferase activities were significantly increased in the MDA-MB-231 and BT549 cells treated with ENC1-P relative to the cells transfected with vector, and ENC1-E2 had the highest activity among ENC1-E1, ENC1-E2, ENC1-E3 and ENC1-E4 versus ENC1-P (Fig. 4B). To further investigate the biological significance of ENC1-SE^distal^, we used the CRISPR-Cas9 nuclease system for the construction of the ENC1-SE^distal^-deleted MDA-MB-231 and BT549 cell lines using a pair of sgRNAs to knock out approximately 9 kb of ENC1-SE internal sequence (E1-E4) (Fig. 4C). We found that SE-deletion had a significant reduction in ENC1 mRNA and protein expression (Fig. 4D, E). Of note, we found no significant changes in the expression of HEXB and microRNA-544 on the same chromosome after SE-deletion (Fig. 4F). More importantly, SE-deletion in RaR cells significantly increased the sensitivity of RaR cells to radiotherapy (Supplementary Fig. S2A–C). The above results can illustrate that aberrant SE-driven regulation of ENC1 can promote the resistance of BC cells to radiotherapy.

### SE-associated ENC1 is transcriptionally activated by TCF4

**Fig. 5:**
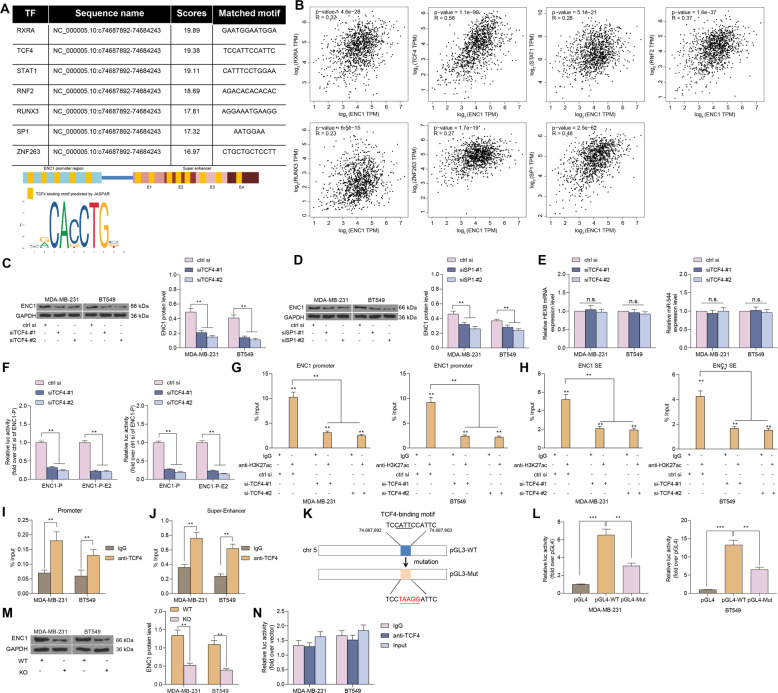
SE-associated ENC1 is transcriptionally activated by TCF4. **A** Transcription factors that bind to the ENC1-P as well as the SE regions predicted by the JASPAR website (upper) and binding sites of TCF4 to ENC1-P and ENC1-SE and conserved binding sequences of TCF4 predicted by JASPAR website (down). **B** Correlations between expression of seven transcription factors and ENC1 expression in the TCGA-BRCA database analyzed by Spearman’s correlation test. **C**, **D** The ENC1 expression in MDA-MB-231 and BT549 after knocking down the expression of TCF4 (**C**) and SP1 (**D**) determined by western blot. **E** The expression of HEXB and miR-544, located in the same chromosomal region of ENC1, in MDA-MB-231 and BT549 cells after knockdown of TCF4 or SP1 determined by RT-qPCR. **F** Detection of luciferase activity in ENC1-P and ENC1-P-SE2 in MDA-MB-231 and BT549 cells after knockdown of TCF4. **G**, **H** The level of H3K27ac modifications in the ENC1-P (**G**) and ENC1-E2 (**H**) after knockdown of TCF4 determined by ChIP-qPCR. **I**, **J** The binding relationship between TCF4 and ENC1-P (**I**) and ENC1-E2 (**J**) examined by ChIP. **K** schematic representation of a 241 bp region of the ENC1 enhancer (from chr5: 74687892-74684243) containing the TCF4-wt motif binding sequence or the mut alleles. **L** BC cells were transfected with the plasmids for 2 d. The luciferase activity levels were normalized to pGL4 luciferase activity. **M** After constructing cell lines with ENC1-P and ENC1-SE knock out using the Crispr-Cas9 system, the expression of ENC1 in cells was detected by western blot. **N** ChIP-qPCR in MDA-MB-231 and BT549 cells with ENC1-P and ENC1-SE knock out using anti-TCF4 antibody. Data are representative of 3 separate experiments performed in triplicate. All the data are expressed as the mean ± SD. Two-way ANOVA with Tukey’s multiple comparison test were utilized to detect significant differences between data. **p < 0.01, ***p < 0.001, ns, nonsignificant.

We further investigated how SE regulates ENC1 expression, thereby influencing the growth of BC cells in vitro and radio-resistance in vivo. Since enhancers are regulatory DNA elements comprised of transcription factor binding sites, they are uniquely able to stimulate transcription over large genomic distances [31]. Therefore, we aimed to identify transcription factors capable of activating the ENC1-SE. So, we analyzed the transcription factors that could bind to the promoter of ENC1 as well as the E2 enhancer in JASPAR (http://jaspar.genereg.net/), and we screened a total of seven transcription factors (Fig. 5A) by setting Scores > 18.0 as the threshold. We further examined the correlation between the expression of these seven transcription factors and ENC1 expression in the TCGA database. The expression of TCF4 and SP1 had the highest correlation with ENC1 expression under Spearman’s correlation analysis (Fig. 5B). To test our conjecture, we knocked down the expression of TCF4 and SP1 in BC cells. We found that knockdown of both SP1 and TCF4 could reduce the expression of ENC1 in cells, and it was worth noting that the suppressing effects on ENC1 expression was more pronounced in cells transfected with si-TCF4 (Fig. 5C, D). Moreover, knocking down TCF4 expression did not affect the expression of HEXB and miR-544 (Fig. 5E).

To further verify that TCF4 could directly promote ENC1 expression transcriptionally by SE, we tested whether TCF4 could mediate the luciferase activity of the ENC1-P and ENC1-E2. Consistent with our conjecture, the luciferase activity of the ENC1-P and ENC1-E2 was significantly reduced after knockdown of TCF4 (Fig. 5F). We subsequently performed ChIP-qPCR experiments using H3K27ac antibodies, which revealed that si-TCF4 reduced the level of H3K27ac modification (Fig. 5G, H) in ENC1-P and ENC1-E2. And, we performed additional ChIP-qPCR experiments using TCF4 or IgG antibodies, which showed that TCF4 was strongly correlated with the ENC1-P and ENC1-E2 (Fig. 5I, J). Based on the results of our Fig. 5A, we designed wild-type (pGL4-WT) and mutant (pGL4-MT) luciferase reporter vectors (Fig. 5K) based on the TCF4 binding site to ENC1 (TCCATTCCATTC). The luciferase activity of pGL4-WT was significantly increased in MDA-MB-231 and BT549 cells, whereas the luciferase activity of pGL4-MT transfected cells had a significant decrease (Fig. 5L). To further investigate the binding relationship between TCF4 and ENC1 promoter with Enhancer, we used Crispr-Cas9 to knock out the promoter of ENC1 together with the enhancer region in MDA-MB-231 and BT549 cells. RT-qPCR revealed that the expression of ENC1 in cells was significantly reduced (Fig. 5M). Moreover, the results of ChIP-qPCR experiments showed that the recruitment of ENC1 promoters and ENC1 enhancer by anti-TCF4 did not change significantly compared with IgG (Fig. 5N). The above results indicate that TCF4 has a significant promoting effect on SE activity.

### Abnormal SE-driven ENC1 mediated by TCF4 correlates with poor prognosis in BC patients and radio-resistance

**Fig. 6:**
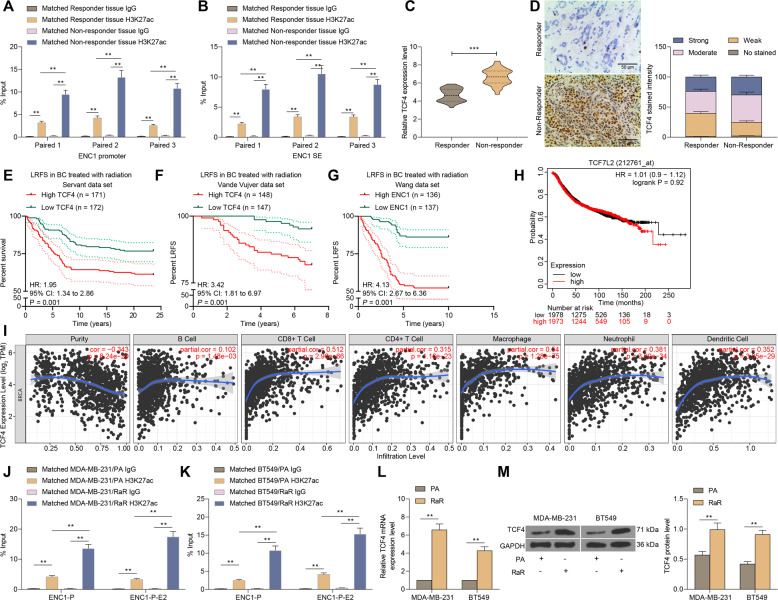
SE-driven ENC1 mediated by TCF4 correlates with poor prognosis in BC patients and radio-resistance. **A**, **B** ChIP analysis of three pairs of breast tissues from non-responders and responders using anti-H3K27ac antibodies. qPCR was used to quantify H3K27ac levels in the ENC1-P (**A**) and ENC1-E2 (**B**). **C**, **D** Expression of TCF4 by RT-qPCR (**C**) and immunohistochemistry (**D**) in 39 non-responders and 52 responders. **E**–**G** Correlation between TCF4 expression and LRFS in BC patients in Servant (**E**), Vande Vijver (**F**), and Wang (**G**) datasets. **H** Analysis of the association between TCF4 expression and survival of patients in TCGA-BRCA dataset. **I** analysis of the relationship between TCF4 expression and the infiltration of immune cells in BC patients. **J**, **K** ChIP-qPCR analysis was used to quantify H3K27ac levels in the ENC1-P and ENC1-E2 in MDA-MB-231 (**J**) and BT549 cells (**K**). **L**, **M** RT-qPCR (**L**) and western blot (**M**) detection of the expression of TCF4 at mRNA and protein levels in cells. Data are representative of 3 separate experiments performed in triplicate. All the data are expressed as the mean ± SD. Unpaired *t* test, one-way or two-way ANOVA with Tukey’s multiple comparison test were utilized to detect significant differences between data. **p < 0.01, ***p < 0.001.

First, we detected H3K27ac levels at the ENC1-P (Fig. 6A) and ENC1-E2 (Fig. 6B) in responders and non-responders. Consistent with our conjecture, the results of the ChIP experiments demonstrated significantly higher levels of H3K27ac at the ENC1-P and ENC1-E2 in non-responder tumor tissues than those in the responder tumor tissues. Moreover, TCF4 was expressed at much higher levels in non-responders than responders (Fig. 6C, D). We further analyzed LRFS in the BC datasets Servant, Vande Vijver, and Wang. The patients with poor expression of TCF4 had higher survival after radiotherapy in the three datasets (Fig. 6E–G). However, we found no significant correlation between TCF4 expression and the overall survival rates in patients in the TCGA-BRCA database (Fig. 6H). Still, we further analyzed the relationship between TCF4 expression and immune cell infiltration in patients in the TCGA-BRCA database. Significant positive correlation between TCF4 expression and infiltration of CD8^+^ T cells, macrophages, neutrophils, and dendritic cells were noted (Fig. 6I).

In addition, the level of H3K2ac modification in the ENC1-P and ENC1-E2 was significantly increased in RaR cells relative to PA cells (Fig. 6J, K). Moreover, we further found that the expression of TCF4 was significantly lower in PA cells than in RaR cells (Fig. 6L, M). The above results indicate that the overexpression of TCF4 in BC tissues promotes H3K27ac modification in the ENC1-P and ENC1-E2, thereby elevating ENC1 expression and conferring radio-resistance in BC.

### Overexpression TCF4 abrogates radio-sensitivity induced by siENC1

To further clarify the effect of TCF4 in BC cells resistance to radiotherapy, we overexpressed TCF4 in MDA-MB-231/RaR and BT549/RaR cells with poor expression of ENC1, or knocked down TCF4 expression in MDA-MB-231/PA and BT549/PA cells overexpressing ENC1. A significant augment in the expression patterns of ENC1 after overexpression of TCF4 in MDA-MB-231/RaR and BT549/RaR cells and a significant decrease in the expression of ENC1 after silencing of TCF4 in MDA-MB-231/PA and BT549/PA cells were observed (Supplementary Fig. S3A). Moreover, we analyzed the radio-sensitivity of PA and RaR cells. MDA-MB-231/PA and BT549/PA cells demonstrated reduced resistance to radiotherapy after knocking down TCF4, MDA-MB-231/RaR and BT549/RaR cells displayed diminished sensitivity to radiotherapy following TCF4 overexpression (Supplementary Fig. S3B, C).

Subsequently, the results of our in vivo experiments showed that after further knockdown of TCF4 expression in MDA-MB-231/PA and BT549/PA with high expression of ENC1, MDA-MB-231 and BT549 cells had significantly reduced metastatic capacity in vivo (Supplementary Fig. S4A); the survival of mice was significantly increased (Supplementary Fig. S4B). Also, we found that overexpression of TCF4 significantly promoted the resistance of RaR cells to radiotherapy in vivo and enhanced tumor growth rate (Supplementary Fig. S4C, D). We found that the protein expression of TCF4 and ENC1 in tumor tissues was largely consistent with those in cells (Supplementary Fig. S4E). Moreover, overexpression of TCF4 reduced the apoptosis rate of tumor tissues after radiotherapy in the presence of si-ENC1 (Supplementary Fig. S4F).

To further clarify the effect of TCF4 on radiotherapy resistance in BC cells, we knocked down TCF4 in MDA-MB-231/RaR cells. Knockdown of TCF4 had a significant decrease in the expression of ENC1 in cells, but further overexpression of ENC1 in cells increased the protein expression of ENC1 in cells (Supplementary Fig. S5A). Subsequently, knockdown of TCF4 significantly promoted the radiosensitivity of MDA-MB-231/RaR cells, but further overexpression of ENC1 significantly inhibited this effect (Supplementary Fig. S5B). Also, under 2 Gy irradiation, apoptosis was significantly increased in cells with poor TCF4 expression, but decreased by overexpression of ENC1 (Supplementary Fig. S5C).

### ENC1 promotes radio-resistance in BC cells through activation of the Hippo pathway

**Fig. 7:**
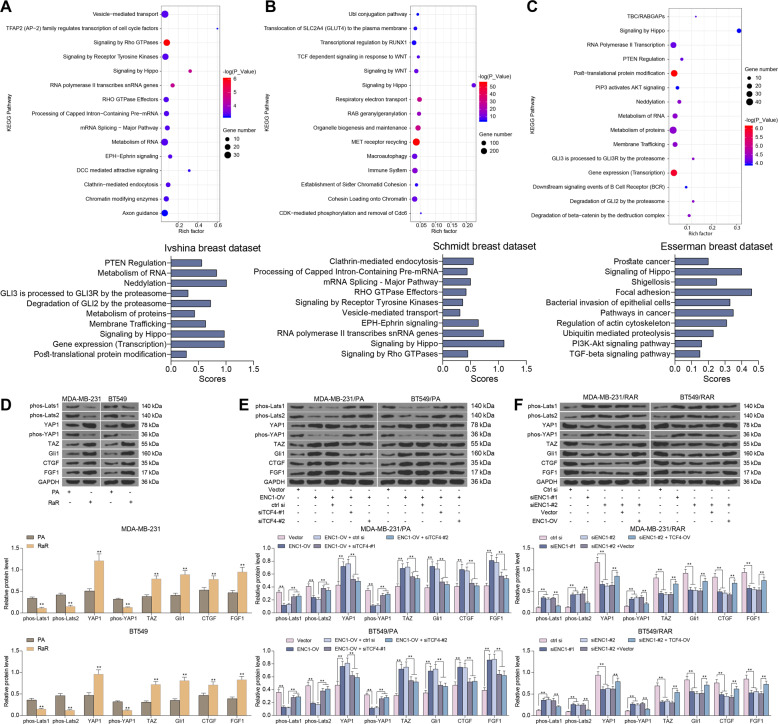
ENC1 promotes radio-resistance in BC cells by activating the Hippo signaling pathway. **A**–**C** Ivshina breast cancer dataset (**A**), the Schmidt breast dataset (**B**), and the Esserman breast dataset (**C**) were accessed from the Oncomine database, and correlation analyses with ENC1 expression were performed to collect Spearman correlation coefficients. Genes greater than 0.45 were subjected to reactome signaling pathway enrichment analysis. **D**–**F** Western blot detection of phosphorylation levels of Lasts 1/2, YAP1 and TAZ, and expression of Gli1, CTGF and FGF1 in untreated MDA-MB-231 and BT549 cells (**D**), MDA-MB-231/RA and BT549/RA cells (**E**) and MDA-MB-231/RaR and BT549/RaR cells (**F**). Data are representative of 3 separate experiments performed in triplicate. All the data are expressed as the mean ± SD. Two-way ANOVA with Tukey’s multiple comparison test were utilized to detect significant differences between data. **p < 0.01.

To clarify the regulatory signaling pathway of ENC1, we accessed the Ivshina breast cancer dataset [18], the Schmidt breast dataset [19], and the Esserman breast dataset [20] from the Oncomine database, respectively. The data were then subjected to correlation analyses with ENC1 expression, and genes with Spearman correlation coefficients greater than 0.45 were collected for reactome signaling pathway enrichment analysis, respectively. We found that the Hippo signaling pathway was enriched in all three datasets (Fig. 7A–C). Subsequently, we further analyzed the levels of Hippo signaling pathway-related markers, and the results of western blot experiments were consistent with what we suspected. Significantly decreased levels of phosphorylated Last1/2 and phosphorylated YAP1 and TAZ, along with remarkably elevated expression of the anti-apoptotic and pro-proliferatory genes Gli1, CTGF and FGF1 were observed in RaR cells. However, after further silencing of ENC1 expression in RaR cells, the phosphorylated levels of Lasts1/2, YAP1 as well as TAZ increased notably, and the expression of downstream genes decreased significantly, but overexpression of ENC1 in the PA cells had completely opposite experimental results (Fig. 7D–F).

**Fig. 8:**
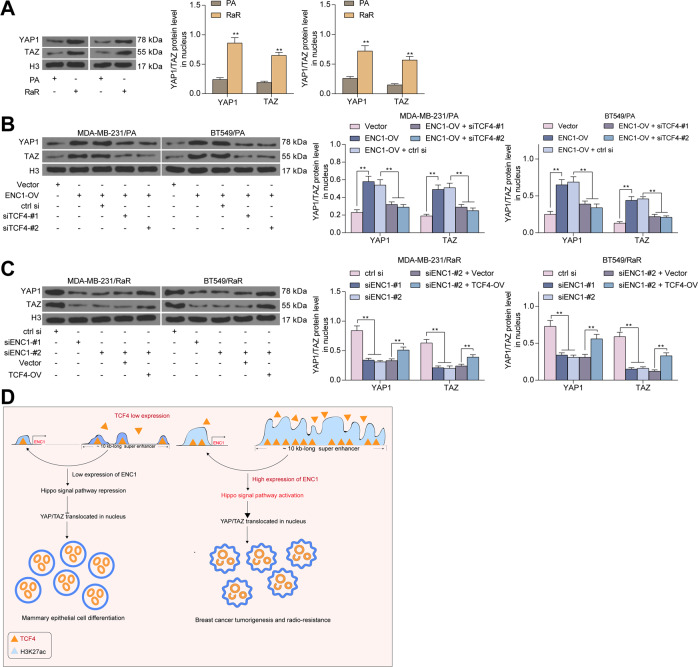
ENC1 promotes the nuclear translocation of YAP1 with TAZ. **A**–**C** Western blot detection of YAP1 and TAZ nuclear translocation in untreated MDA-MB-231 and BT549 cells (A), MDA-MB-231/RA and BT549/RA cells (**B**) and MDA-MB-231/RaR and BT549/RaR cells (**C**). **D** ENC1 is driven by SE in BC, thereby promoting radio-resistance in BC cells. Data are representative of 3 separate experiments performed in triplicate. All the data are expressed as the mean ± SD. Two-way ANOVA with Tukey’s multiple comparison test were utilized to detect significant differences between data. **p < 0.01.

Furthermore, we examined the expression of YAP1 and TAZ in the nucleus, as the translocation of YAP1 and TAZ complexes into the nucleus is one of the methods by which the Catholic Hippo pathway exercises its function. The expression of YAP1 and TAZ in the nucleus were notably increased in RaR cells, but further reduction of ENC1 significantly decreased the expression of YAP1 and TAZ in the nucleus. However, overexpression of ENC1 in the PA cells significantly promoted the YAP1 and TAZ nuclear translocation (Fig. 8A–C). Consistent results were also found in MCF10A and HB-2 cells after overexpression of ENC1 (Supplementary Fig. S6A, B).

In summary, we concluded that ENC1 is driven by a SE in BC. We found a ~10-kb long SE located 60 kb~ upstream of ENC1, responsible for driving ENC1 expression and its function in radio-resistance. We also demonstrated that increased expression of TCF4 leads to aberrant activity of the SE^distal^ regulating ENC1 expression in BC, thereby promoting the nuclear translocation of YAP1 and TAZ in the Hippo signaling pathway and the expression of the downstream anti-apoptotic genes Gli1, CTGF and FGF1 to enhance radio-resistance in BC cells (Fig. 8D).

## Discussion

BC is the most frequent malignancy in the female population, and one of the three most frequently-diagnosed cancers worldwide, just after lung and colon cancers [32]. The only treatment regimens remain chemo- and/or radio-therapy in addition to surgery, and new biomarkers or treatment options are urgently necessary to improve outcomes for sufferers [33]. SE were identified as key oncogenic drivers in many tumor cells, suggestive of the discovery of cancer therapeutics directed at components of SE in BC [34]. Our analysis revealed that ENC1 represents a mRNA most outstandingly overexpressed in BC tissues. Additionally, investigation of clinical samples of BC patients showed that ENC1 expression correlates closely with metastatic recurrence and poor survival in BC patients. Moreover, ENC1 evidently induces MCF10 and HB-2 cells to invade and migrate, and ENC1 silencing causes dramatic declines in the radio-resistance of the MDA-MB-231 and BT549 BC cells. Therefore, we might provide novel insights into this area of research by recognizing ENC1 as promoter of metastasis in BC.

ENC1 has been previously detected to be higher in serum of male BC than that to healthy individuals [35], while its specific role in BC remains basically unclear. ChIP followed by sequencing conducted by Raisner et al. identified over 2500 unique SE acquired by BC cells, which are absent from normal breast tissues [36]. More specifically, Betancur et al. showed that a large repertoire of active constituent enhancers, located within the two CD47 SEs, modulate CD47 expression in cancer cell types, and that impairment of CD47 SEs reduced CD47 gene expression in BC [37]. Likewise, suppression of SE downregulated the expression of Krüpple-like factor 5 in basal-like BC [38]. Interestingly, we identified the ENC1-SE^distal^ and divided it into four parts (ENC1-E1-4), among which ENC1-E2 exhibited the highest luciferase activity in BC cells. SE-derived noncoding RNAs play vital roles in tumorigenesis, involving malignant proliferation, metastasis and drug resistance [39]. We found that SE deletion in MDA-MB-231/RaR and BT549/RaR cells contributed to promoted radio-sensitivity.

Even though enhancers are recognized to be linked to certain histone modifications and transcription factors, the interrelationship of these alterations to gene expression has not been well-defined [40]. Using the bioinformatics website JASPAR, we predicted seven transcription factors that may bind to ENC1. The subsequent correlation analyses using TCGA database revealed that TCF4 and SP1 shared relatively high correlation coefficients. The following western blot verified that only si-TCF4 resulted in declines in ENC1 expression. Active enhancer regions are characteristically enriched with posttranslational modification histone marks, including H3K27ac [41]. Our ChIP-qPCR assay using antibodies against H3K27ac provided that si-TCF4 downregulated the levels of H3K27ac in both ENC1-P and ENC1-E2. Similarly, the promoter activity of ENC1 has been established to be elevated about 3-fold after transfection of beta-catenin with wild-type TCF4 in HeLa cells [42]. Our following rescue experiments also corroborated that overexpression of TCF4 conferred the radio-resistance to BC cells overcame by siENC1.

Reactome signaling pathway enrichment analysis revealed that the Hippo signaling pathway is enriched in all three datasets, including Ivshina breast cancer [18], chmidt breast [19] and Esserman breast [20] datasets. Moreover, miR-591 serves as an inhibitor in BC by targeting TCF4 and inhibiting the Hippo-YAP/TAZ signaling pathway [43]. The Hippo-YAP/TAZ pathway has also been reported to mediate geranylgeranylation signaling in BC progression [44]. Therefore, we postulated that ENC1 is involved in radio-resistance in BC through the Hippo-YAP/TAZ signaling pathway as well. YAP/TAZ are principally mediated by phosphorylation and suppression of YAP/TAZ transcriptional activity, and two members of the NDR family of kinases, Last1 and 2 are main regulators of YAP/TAZ [45]. In addition,﻿ overexpression of TCF4 induced the expression of Smo and Gli1, which in turn governs fibroblast proliferation [46]. Consistently, we observed that ENC1 overexpression led to the Hippo-YAP/TAZ signaling pathway activation, as evidenced by lowered Last1/2 phosphorylated level, enhanced YAP and TAZ nuclear translocation as well as promoted Gli1, CTGF and FGF1 expression, and silencing of TCF4 resulted in the pathway deficit.

All in all, we found that SE-driven ENC1 expression translationally activated by TCF4 promotes recurrence and radio-resistance of BC cells by simultaneously repressing the extent of Last1/2, YAP1 and TAZ phosphorylation of Hippo signaling. These findings may provide clinically beneficial evidence for exploring a novel prognostic biomarker and therapeutic target for BC radio-resistance.

## Supplementary information

Supplementary Figures

Supplementary Tables

## Data Availability

All the data generated or analyzed during this study are included in this published article.
