# Supplementary material for: Aberrant super-enhancer-driven oncogene ENC1 promotes the radio-resistance of breast carcinoma
Source: Cell Death Dis. 2021 Aug 6;12(8):777. doi: 10.1038/s41419-021-04060-5 (PMC8346480; doi:10.1038/s41419-021-04060-5)
Supplement: Supplementary file 1 — Supplementary Figures [file 41419_2021_4060_MOESM1_ESM.docx]

**Supplementary Fig S1.**


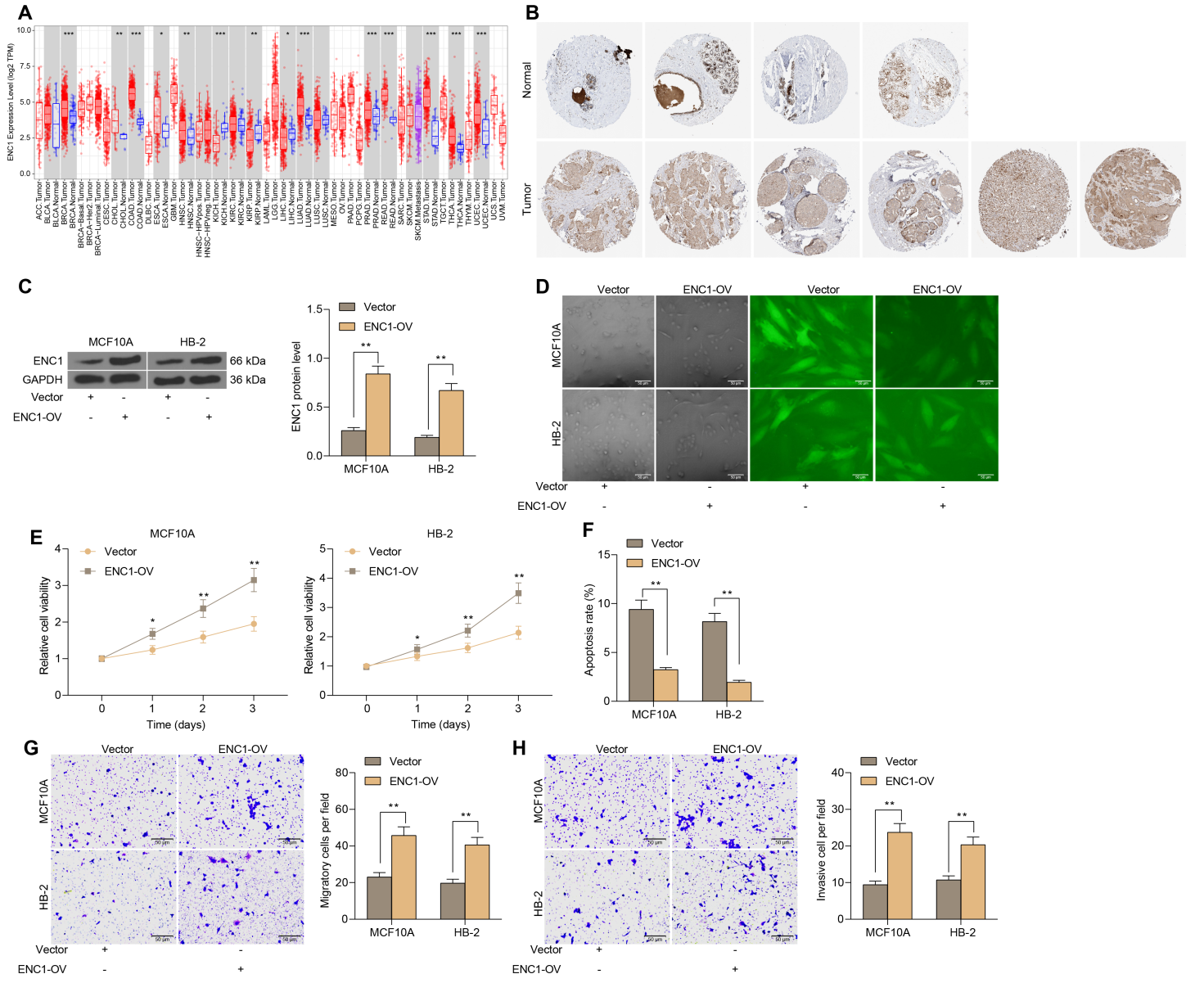


**Supplementary Fig S1.** ENC1 overexpression promotes malignant behavior of breast epithelial cells. A, ENC1 is highly expressed in many cancers; B, ENC1 staining intensity in BC tissues and normal breast tissues in the Human protein atlas database. ENC1-OE or control Vector was delivered into normal mammary epithelial cell lines MCF10A and HB-2 cells. C, transfection efficiency of ENC1-OE or control Vector in MCF10A and HB-2 cells determined by western blot; D, morphological changes of MCF10A and HB-2 cells after overexpression of ENC1 under a light microscopy and the transfection efficiency of plasmid observed under a fluorescence microscopy; E, the proliferative capacity of MCF10A and HB-2 cells determined by CCK-8 assay; F, apoptosis rate of MCF10A and HB-2 cells measured by flow cytometry; G-H, migration and invasion capacity of MCF10A and HB-2 cells determined by Transwell assay. Data are representative of 3 separate experiments performed in triplicate. All the data are expressed as the mean ± SD. Two-way ANOVA with Tukey’s multiple comparison test were utilized to detect significant differences between data. ***p* < 0.01.

**Supplementary Fig S2.**


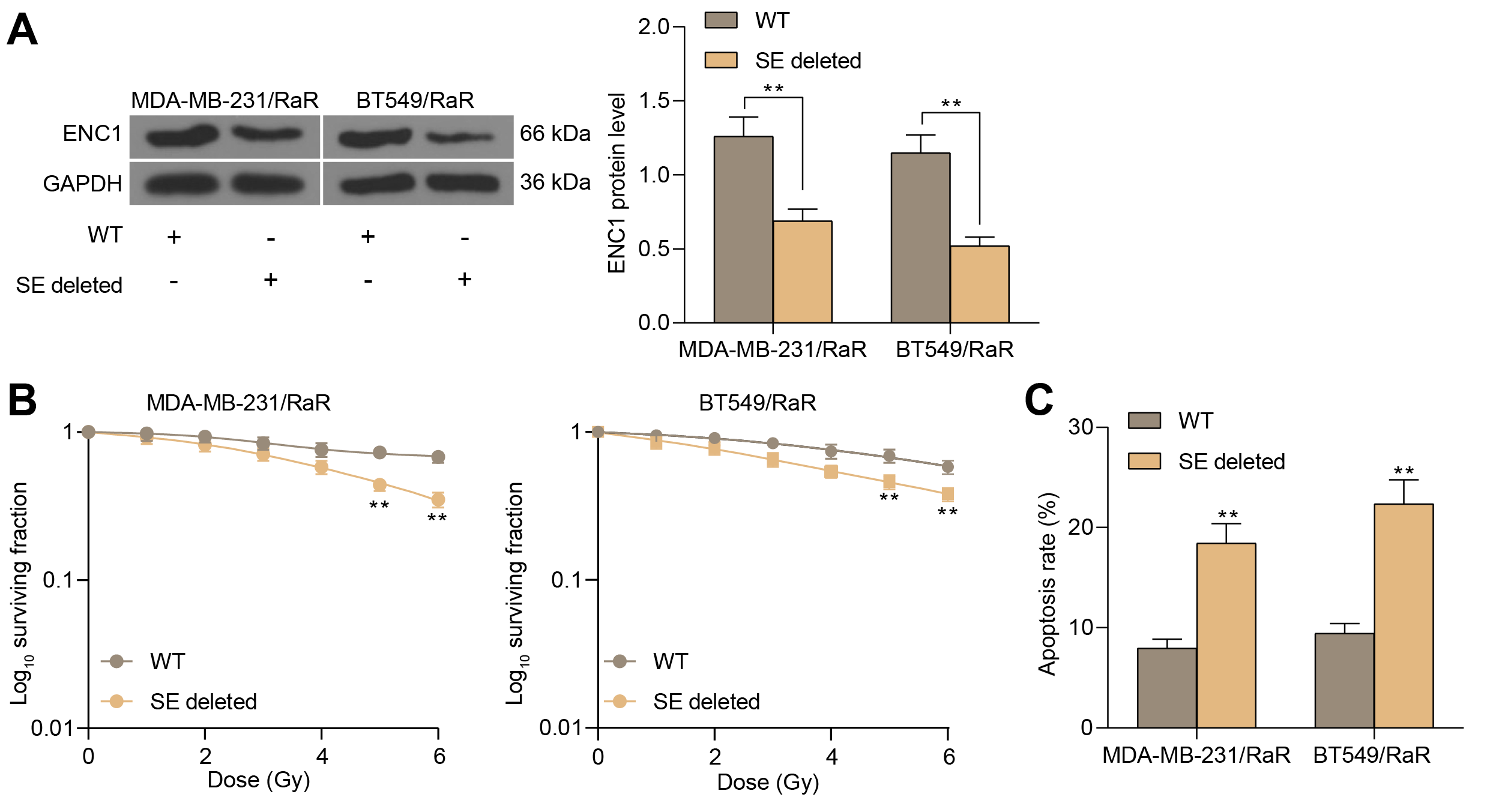


**Supplementary Fig S2.** Deletion of the SE region of ENC1 by CRISPR/Cas9 inhibits radio-resistance in BC cells. A, western blot detection of protein expression of ENC1 in MDA-MB-231/RaR and BT549/RaR cells after SE deletion; B, cell viability of MDA-MB-231/RaR and BT549/RaR cells after 2 h of exposure to different doses of gamma radiations determined by colony formation assay; C, proportion of apoptotic cells detected by flow cytometry after irradiation of MDA-MB-231/RaR and BT549/RaR cells with 2 Gy doses of gamma radiation. Data are representative of 3 separate experiments performed in triplicate. All the data are expressed as the mean ± SD. Two-way ANOVA with Tukey’s multiple comparison test were utilized to detect significant differences between data. ***p* < 0.01.

**Supplementary Fig S3.**

**
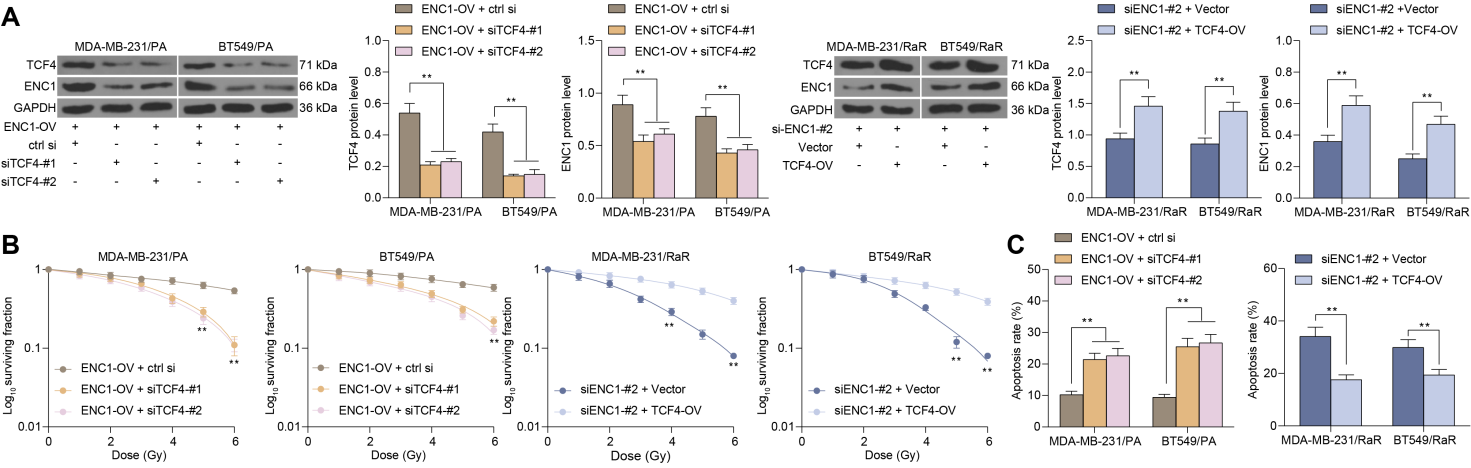
**

**Supplementary Fig S3.** Overexpression TCF4 abrogates radio-sensitivity conferred by siENC1 *in vitro*. TCF4 was overexpressed in MDA-MB-231/RaR and BT549/RaR cells with low expression of ENC1, or knocked-down in MDA-MB-231/PA and BT549/PA cells overexpressing ENC1. A, western blot detection of TCF4 and ENC1 expression in PA or RaR cells; B, cell viability of MDA-MB-231 and BT549 cells after 2 h of exposure to different doses of gamma rays examined by colony formation assay; C, proportion of apoptotic cells detected by flow cytometry after irradiation of PA and RaR cells with 2 Gy doses of gamma. Data are representative of 3 separate experiments performed in triplicate. All the data are expressed as the mean ± SD. Two-way ANOVA with Tukey’s multiple comparison test were utilized to detect significant differences between data. ***p* < 0.01.

**Supplementary Fig S4.**

**
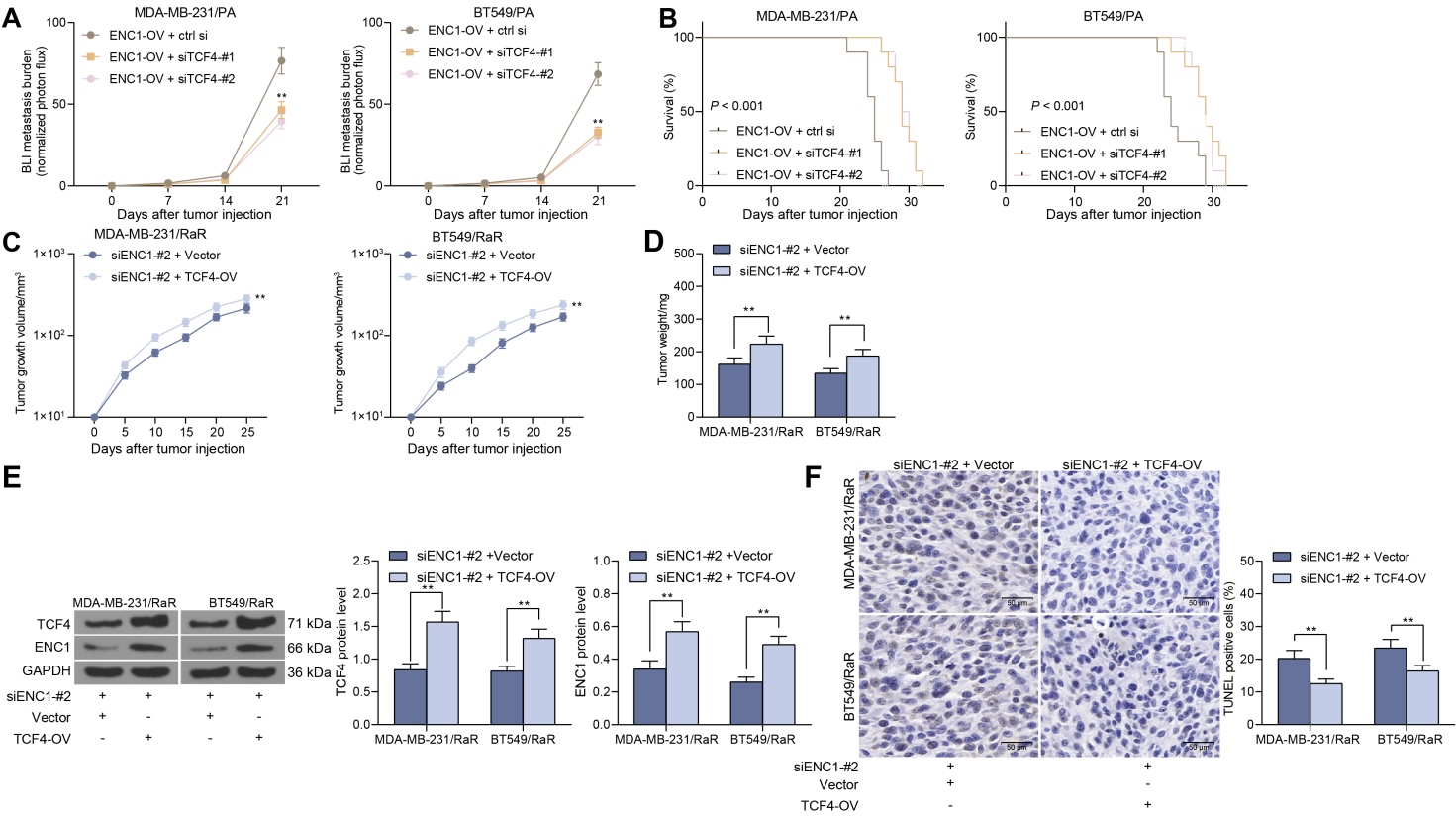
**

**Supplementary Fig S4.** Overexpression TCF4 abrogates radio-sensitivity conferred by siENC1 *in vivo*. A, the metastatic ability of MDA-MB-231/PA and BT549/PA cells overexpressing ENC1 in mice (n = 6); B, analysis of the survival of mice after injection of MDA-MB-231 and BT549 cells (n = 10); C-D, tumor growth curve (C) and weight (D) of nude mice injected with MDA-MB-231 and BT549 cells with ENC1 knockdown (n = 6); E, TCF4 and ENC1 expression in tumor tissues examined using western blot. F, apoptosis rate in tumors examined by TUNEL staining. Data are representative of 3 separate experiments performed in triplicate. All the data are expressed as the mean ± SD. Two-way ANOVA with Tukey’s multiple comparison test were utilized to detect significant differences between data. ***p* < 0.01.

**Supplementary Fig S5.**

**
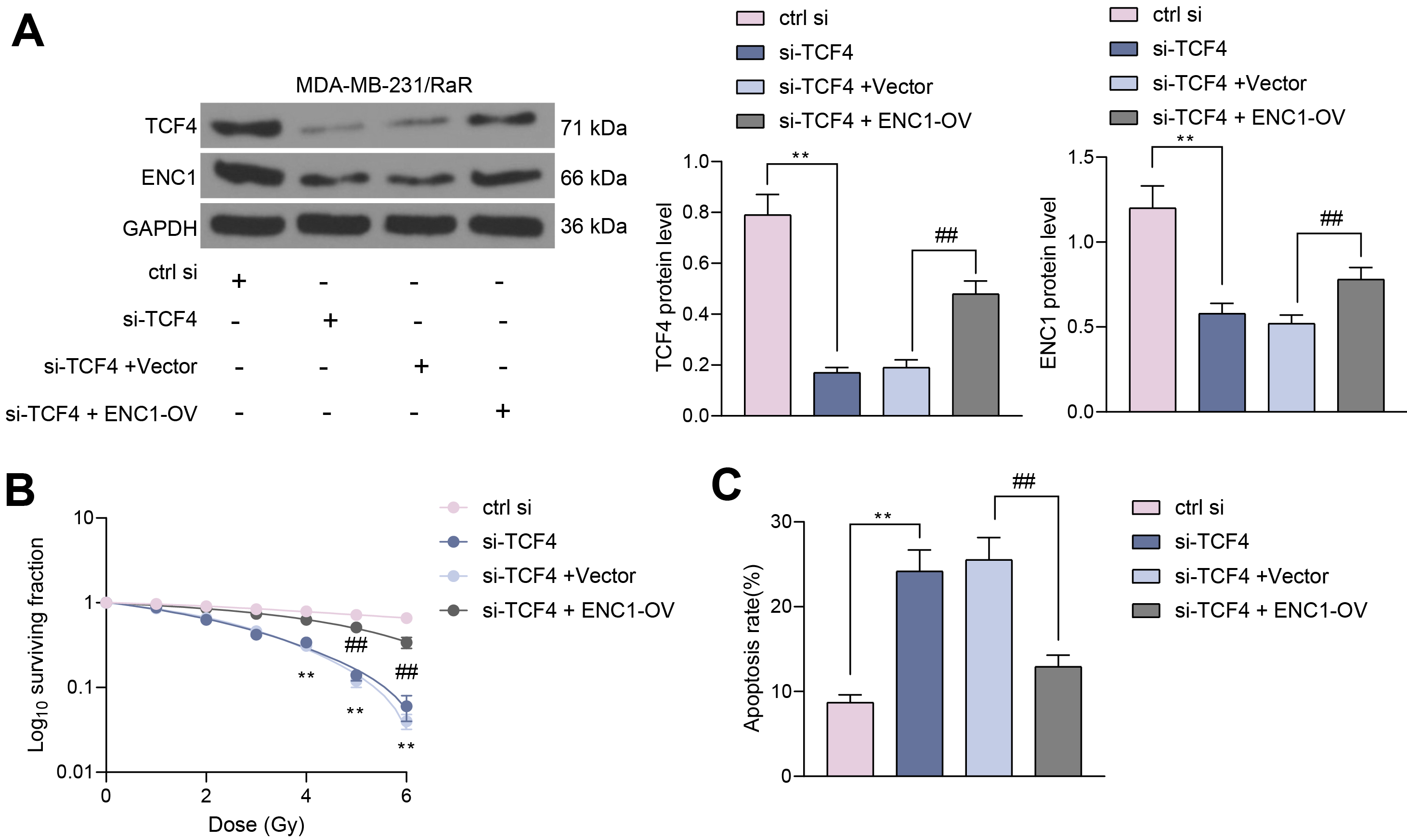
**

**Supplementary Fig S5.** TCF4 silencing induces BC cells radio-sensitivity. MDA-MB-231/RaR cells were transfected with TCF4 silencing and ENC1 overexpression. A, TCF4 and ENC1 protein expression in MDA-MB-231/RaR cells after co-transfection; B, cell viability of MDA-MB-231/RaR cells after 2 h of exposure to different doses of gamma radiations determined by colony formation assay; C, proportion of apoptotic cells detected by flow cytometry after irradiation of MDA-MB-231/RaR cells with 2 Gy doses of gamma radiation. Data are representative of 3 separate experiments performed in triplicate. All the data are expressed as the mean ± SD. Two-way ANOVA with Tukey’s multiple comparison test were utilized to detect significant differences between data. ***p* < 0.01; ##*p* < 0.01.

**Supplementary Fig S6.**


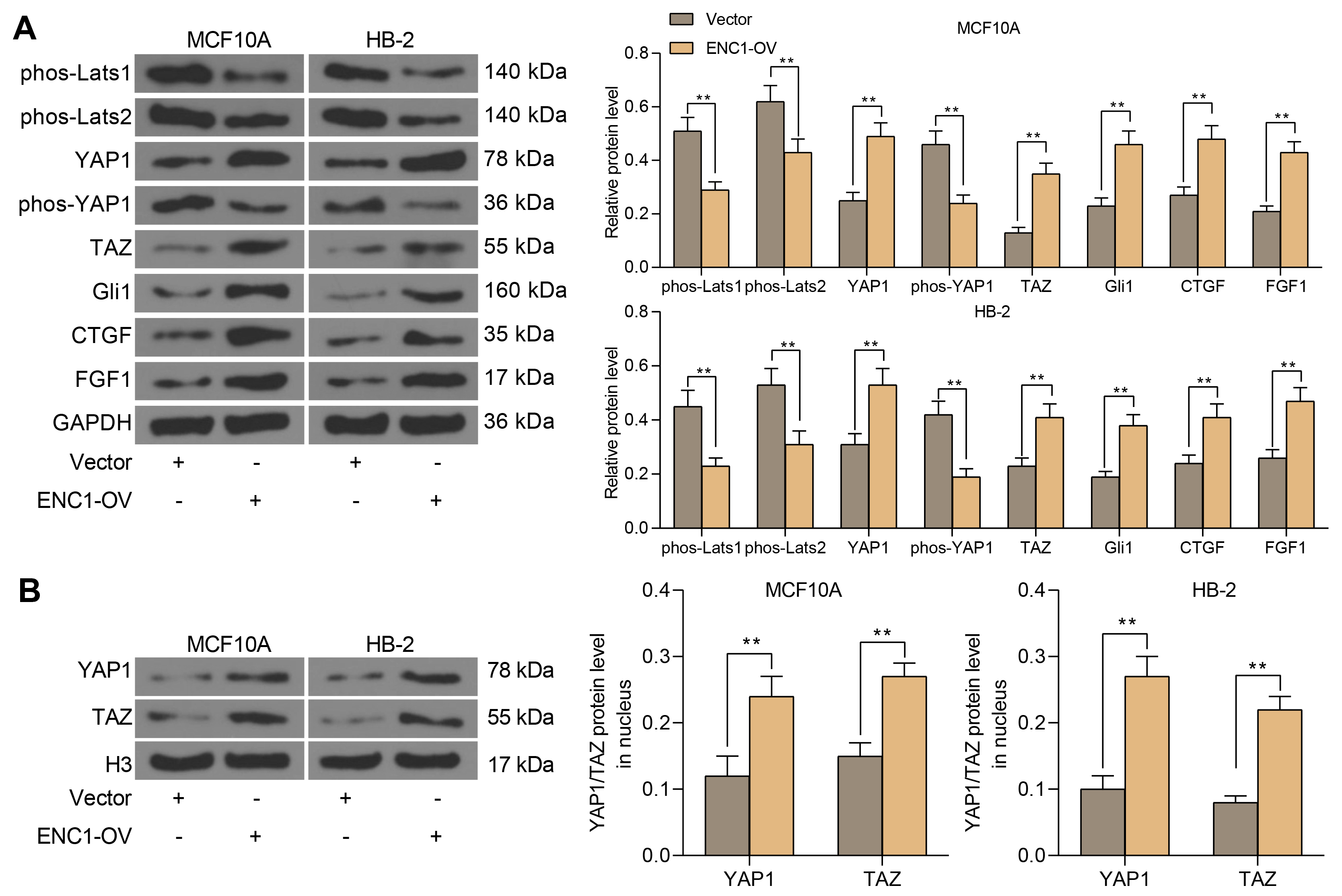


**Supplementary Fig S6.** ENC1 overexpression promotes activation of the Hippo pathway in breast epithelial cells. A, western blot detection of expression of Hippo signaling pathway-related proteins and its downstream proteins in MCF10A and HB-2 cells after overexpression of ENC1; B, western blot detection of YAP1 and TAZ nuclear translocation in MCF10A and HB-2 cells. Data are representative of 3 separate experiments performed in triplicate. All the data are expressed as the mean ± SD. Two-way ANOVA with Tukey’s multiple comparison test were utilized to detect significant differences between data. ***p* < 0.01.
