# Supplementary material for: Aberrant super-enhancer-driven oncogene ENC1 promotes the radio-resistance of breast carcinoma
Source: Cell Death Dis. 2021 Aug 6;12(8):777. doi: 10.1038/s41419-021-04060-5 (PMC8346480; doi:10.1038/s41419-021-04060-5)
Supplement: Supplementary file 2 — Supplementary Tables [file 41419_2021_4060_MOESM2_ESM.docx]

**Supplementary Table S1** Sequence of primers used for real-time PCR

| Symbol | Forward (5’-3’) | Reverse (5’-3’) |
| --- | --- | --- |
| ENC1 | AGACGTGTGGAACAGCATCACC | CATATTATCTCATCGAGTGATGGAG |
| HEXB | GATCCATTGTCTGGCAGGAGGT | GGAAGCCAGATGCTGTGACTCT |
| miR-544 | TCTGCATTTTTAGCAAGTTC | GAACATGTCTGCGTATCTC |
| TCF4 | GCCTCTTCACAGTAGTGCCATG | GCTGGTTTGGAGGAAGGATAGC |
| U6 | CTCGCTTCGGCAGCACAT | TTTGCGTGTCATCCTTGCG |
| GAPDH | GTCTCCTCTGACTTCAACAGCG | ACCACCCTGTTGCTGTAGCCAA |

**Note:** ENC1, ectodermal-neural cortex 1; miR, microRNA; HEXB, hexosaminidase B; TCF4, transcription factor 4; GAPDH, glyceraldehyde-3-phosphate dehydrogenase.

**Supplementary Table S2** Antibody used in Western blot

| Symbol | Catalog | Manufacturer | dilution | Validation |
| --- | --- | --- | --- | --- |
| ENC1 | 15007-1-AP | ProteinTech Group | 1:2000 | KO/KD validation |
| TCF4 | 22337-1-AP | ProteinTech Group | 1:4000 | KO/KD validation |
| Phos-Lats1/2 | Ab111344 | Abcam | 1:5000 | \ |
| YAP1 | GTX129151 | GeneTex | 1:4000 | KO/KD validation |
| Phos-YAP1/TAZ | Ab76252 | Abcam | 1:2000 | \ |
| TAZ | GTX50808 | GeneTex | 1:3000 | Orthogonal validation |
| Gli1 | 66905-1-Ig | ProteinTech Group | 1:4000 | KO/KD validation |
| FGF1 | GTX02554 | GeneTex | 1:6000 | \ |
| GAPDH | 60004-1-Ig | ProteinTech Group | 1:10000 | \ |
| H3 | GTX60888 | GeneTex | 1:2000 | Orthogonal validation |

**Note:** ENC1, ectodermal-neural cortex 1; TCF4, transcription factor 4; FGF1, fibroblast growth factor-1; GAPDH, glyceraldehyde-3-phosphate dehydrogenase; KO, knockout; KD, knockdown.
